# No Added Value of Novel Biomarkers in the Diagnostic Assessment of Patients Suspected of Acute Coronary Syndrome

**DOI:** 10.1371/journal.pone.0132000

**Published:** 2015-07-15

**Authors:** Judith M. Poldervaart, Emma Röttger, Marieke S. Dekker, Nicolaas P. A. Zuithoff, Peter W. H. M. Verheggen, Evelyn A. de Vrey, Thierry X. Wildbergh, Arnoud W. J. van ‘t Hof, Arend Mosterd, Arno W. Hoes

**Affiliations:** 1 Julius Center for Health Sciences and Primary Care, University Medical Center, Universiteitsweg 100, 3584 CG, Utrecht, the Netherlands; 2 Faculty of Medicine, Utrecht University, Universiteitsweg 98, 3584 CG, Utrecht, the Netherlands; 3 Department of Cardiology, Isala Clinics, Dokter van Heesweg 2, 8025 AB, Zwolle, the Netherlands; 4 Department of Cardiology, Meander Medical Center, Maatweg 3, 3813 TZ, Amersfoort, the Netherlands; University of Bologna, ITALY

## Abstract

**Background:**

Despite the availability of high-sensitive troponin (hs-cTnT), there is still room for improvement in the diagnostic assessment of patients suspected of acute coronary syndrome (ACS). Apart from serial biomarker testing, which is time-consuming, novel biomarkers like copeptin have been proposed to expedite the early diagnosis of suspected ACS in addition to hs-cTnT. We determined whether placenta derived growth factor (PlGF), soluble Fms-like tyrosine kinase 1 (sFlt-1), myoglobin, N-terminal prohormone B-type Natriuretic Peptide (NT-proBNP), growth-differentiation factor 15 (GDF-15) and copeptin improved early assessment of chest pain patients.

**Methods:**

This prospective, single centre diagnostic FAME-ER study included patients presenting to the ED with symptoms suggestive of ACS. Blood was collected to measure biomarkers, notably, hs-cTnT was retrospectively assessed. Added value of markers was judged by increase in AUC using multivariable logistic regression.

**Results:**

Of 453 patients enrolled, 149 (33%) received a final diagnosis of ACS. Hs-cTnT had the highest diagnostic value in both univariable and multivariable analysis. PPVs of the biomarkers ranged from 23.5% (PlGF) to 77.9% (hs-cTnT), NPVs from 67.0% (PlGF) to 86.4% (hs-cTnT). Only myoglobin yielded diagnostic value in addition to clinical symptoms and electrocardiography (ECG) (AUC of clinical model 0.80) with AUC of 0.84 (p<0.001). However, addition of hs-cTnT was superior (AUC 0.89, p<0.001). Addition of the biomarkers to our clinical model and hs-cTnT did not or only marginally (GDF-15) improved diagnostic performance.

**Conclusion:**

When assessing patients suspected of ACS, only myoglobin had added diagnostic value beyond clinical symptoms and ECG. However, when combined with hs-cTnT, it yields no additional diagnostic value. PlGF, sFlt-1, NT-proBNP, GDF-15 and copeptin had no added value to the clinical model or hs-cTnT.

## Introduction

The diagnostic assessment of patients suspected of acute coronary syndrome (ACS) remains a challenge. In this diagnostic process, biomarkers play a pivotal role when the electrocardiogram (ECG) is inconclusive. Early diagnosis of ACS is essential because of clear improvement in prognosis following timely interventions, while early ruling out of ACS reduces patient burden and costs. Currently, the definitive diagnosis of ACS is based on elevation of high-sensitive cardiac troponin I or T (hs-cTnI or hs-cTnT), in the context of clinical findings and ECG changes.[1–4] Although high sensitive troponin assays can detect circulating troponins at a lower level in the blood than the previous conventional troponin assays, their diagnostic accuracy is still not considered optimal. To further reduce the number of false-positives and false-negatives, serial testing (usually after three hours) has been suggested, but this is time-consuming and increases health care costs.[5, 6] Alternatively, other biomarkers, some capable of detecting ischemia very soon after symptom onset, have been proposed to be combined with hs-cTn, for example copeptin, which has been advocated in numerous articles.[7–9] Growth differentiation factor-15 (GDF-15) and copeptin are both markers of stress, the former of hemodynamic and the latter of endogenous stress, and are therefore thought to increase even before necrosis occurs.[10, 11] Soluble fms-like tyrosine kinase-1 (sFlt-1) binds placental growth factor (PlGF), a protein that appears to promote the inflammatory process of atherosclerosis and appears to be an early marker of ischemic events.[12] N-terminal prohormone B-type Natriuretic Peptide (NT-proBNP) is a biomarker of myocardial dysfunction and as such reflects the extent of an ischemic insult and its levels correlate with (left) ventricular dysfunction.[13, 14] In addition, we also assessed the diagnostic value of myoglobin, a marker of myocardial necrosis, and known for its rapid rise (<2 hours), but its diagnostic value in combination with hs-cTn has not been fully quantified.[15] Importantly, earlier studies on novel biomarkers mostly focus on the diagnostic characteristics of the biomarker per se, rather than assessing the *added* value of the novel biomarkers to readily available information from medical history, clinical signs and symptoms, and ECG.[16] Moreover, the majority of previous studies evaluated novel biomarkers in both ST-segment elevation myocardial infarction (STEMI) and non-ST-segment elevation myocardial infarction (NSTEMI) patients,[17, 18] while there seems to be no diagnostic dilemma in STEMI patients. The available studies in NSTEMI patients,[13, 19] where additional biomarkers are more urgently needed, exclude patients with unstable angina (UA), while these patients per definition have non-elevated troponins.[1] Since these patients are at increased risk of cardiovascular events or death, novel biomarkers might be very useful to identify these patients. Our aim was to determine whether the novel biomarkers PlGF, sFlt-1, NT-proBNP, GDF-15 and copeptin, as well as myoglobin improve the early diagnosis or exclusion of myocardial infarction or unstable angina, in patients presenting with chest pain at the emergency department (ED), in addition to readily available information from patient characteristics, ECG and hs-cTnT.

## Materials and Methods

### Setting and study population

The FAME-ER (Fatty Acid binding protein in Myocardial infarction Evaluation in the Emergency Room) study was a single centre, prospective diagnostic study among patients presenting to the ED with symptoms suggestive of ACS. After a training period of all professionals involved, all cardiac patients admitted to the ED of the Meander Medical Centre (large regional teaching hospital in Amersfoort, the Netherlands) between May 2007 and November 2007 were identified. Eligible patients were those presenting with symptoms suggestive of ACS within 24 hours of symptom onset. Clear cut ST-segment elevation ACS was an exclusion criterion as these patients underwent primary percutaneous coronary intervention (PCI) elsewhere. Patients of whom no signed informed consent was obtained were excluded. This study complies with the Declaration of Helsinki, furthermore the protocol was approved by the local ethics committee of the Meander Medical Centre.

### Routine clinical assessment

Directly upon presentation to the ED, a standard 12-lead ECG was recorded and venous blood was drawn to determine hs-cTnT, the five novel biomarkers and myoglobin. The plasma component was frozen and stored at -70°C until sample analysis. History taking and physical examination was performed by the ED physician or attending cardiologist. All ECGs were interpreted by the attending cardiologist. Patients were diagnosed and treated according to routine clinical protocols (based on European Society of Cardiology (ESC) guidelines),[1,2] including serial ECGs, and measurement of (high sensitive) troponin.

### Measurement of biomarkers

For information on biomarker assays and cut-off values, we refer to S1 Text.

### Outcomes

The primary outcome of this study was ACS (i.e. STEMI, NSTEMI and UA). The presence of ACS was determined according to the universal definition of myocardial infarction [3, 4] that prevailed at the time of inclusion of participants. A myocardial infarction was defined in accordance with existing guidelines, based on a combination of ischemic symptoms, release of biomarkers of myocardial necrosis (i.e. troponin); with either persistent ST-elevation (STEMI) or no ST-elevation on ECG (NSTEMI). [2, 3, 4] Unstable angina was defined as symptoms associated with dynamic ischemic ECG changes, evidence of ischemia on functional testing or new coronary angiographic changes, without elevation of cTnT. The final diagnosis was made during consensus meetings of an outcome panel (two cardiologists, one resident). The final diagnosis was based on all available clinical information including serial conventional cTnI measurements, a single hs-cTnT measurement, serial ECG findings and hospital discharge letters. Determination of a single hs-cTnT measurement was performed post hoc from the frozen plasma. The outcome panel was blinded to results of the novel biomarkers to prevent incorporation bias.[20]

### Statistical analysis

Continuous variables are presented as means (± standard deviation, SD) or medians (interquartile range, IQR), while categorical variables are presented as numbers (percentage). Comparisons of continuous variables were made with the use of the Mann-Whitney *U*-test. From 2x2 tables, the sensitivity, specificity, and predictive values were calculated. The cut-off values of the biomarkers PlGF (27pg/ml), sFlt-1 (70pg/ml), myoglobin (50ng/ml), NT-proBNP (125pg/ml), GDF-15 (1800pg/ml), and copeptin (14pmol/l) were based on the available literature.[9, 10, 13, 18, 21–23] Receiver operating characteristic (ROC) curves were created and the area under the curve (AUC) was calculated to quantify the diagnostic accuracy of each individual biomarker. Odds ratios (OR) of all possible predictors of ACS were calculated by univariable logistic regression. These predictors were selected based on the literature and clinical experience. From these predictors a clinical model was developed (in part based on their availability at presentation) using the following predictors: patient history (age, sex, previous myocardial infarction, PCI or coronary artery bypass graft (CABG)), cardiovascular risk factors (hypertension, hypercholesterolemia, family history of cardiovascular disease (CVD), smoking, diabetes mellitus) and ECG findings. The diagnostic value of the novel biomarkers in addition to the clinical model, as well as of the clinical model alone, was estimated by using multivariable regression, likelihood ratio’s and ROC curves analyses including the biomarkers as continuous variables.[24] Because of skewed distribution (and linearity) all biomarkers were transformed using natural logarithm. Restricted cubic splines were used to test whether continuous variables had a linear association with the outcome. Discrimination of the multivariable models was determined by the AUC or c-statistic indicating the probability that two patients (one with and one without ACS) are classified correctly.[24] Bootstrapping techniques were used as a validation method to adjust for over-optimism.[25] We performed additional analyses to study whether the diagnostic accuracy differed according to time since onset of symptoms (<3 hours). Multiple imputation techniques were applied in case of missing values.[26] We followed the STARD (Standards for reporting of diagnostic accuracy) checklist.[27, 28] All analyses were performed using Statistical Package for the Social Sciences for Windows 20.0 (SPSS Incl. Chicago, Illinois).

## Results

### Patient characteristics

**Fig 1:**
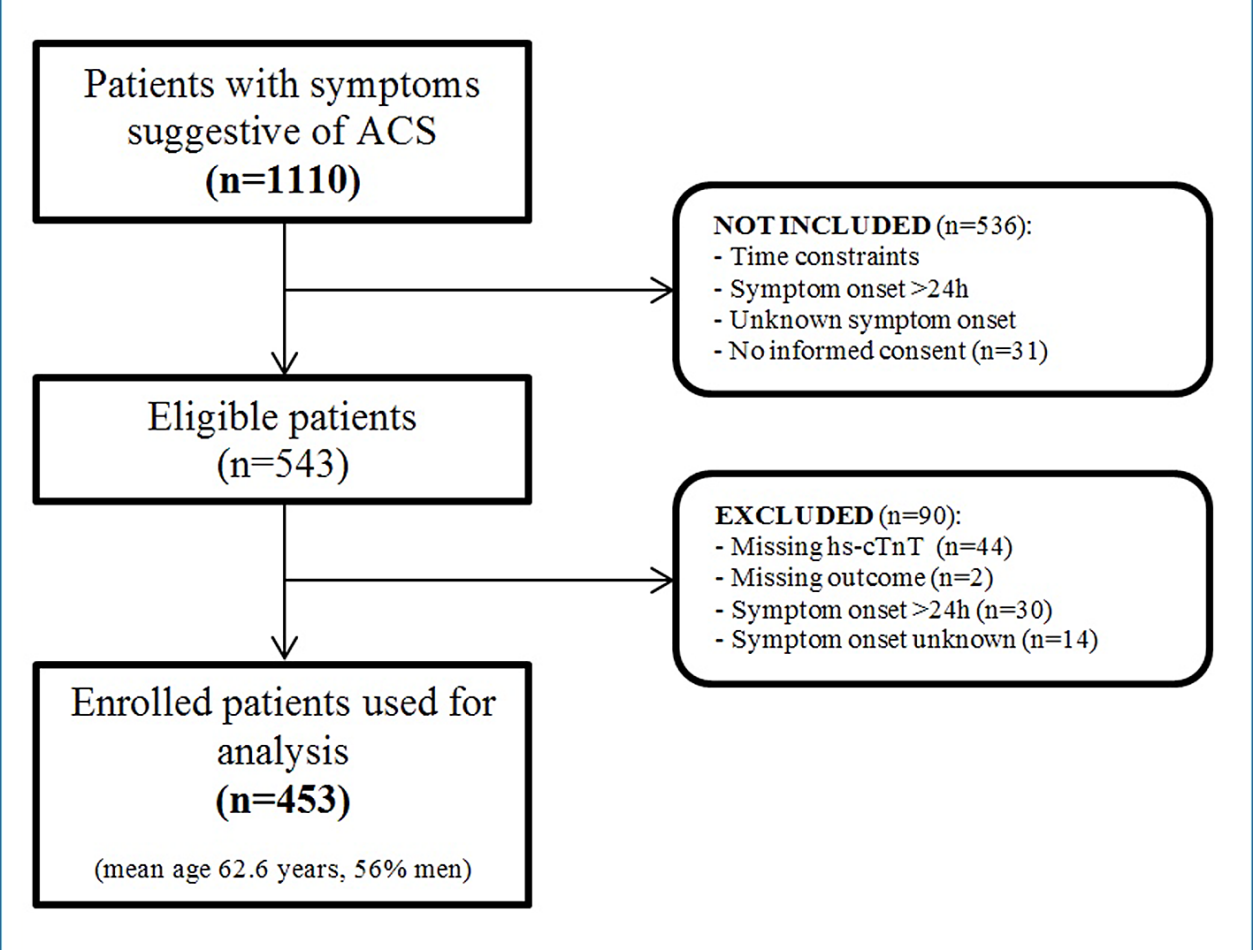
Flow chart of patient selection from all patients with symptoms suggestive of ACS to enrolled patients.

A total of 1110 patients with symptoms suggestive of ACS were identified. Of these, 567 patients were excluded due to time constraints or no obtained informed consent. Another 90 patients were excluded because of major missing values (outcome, hs-cTnT measurements) and/or symptom onset unknown or >24 hours. Eventually, 453 patients were enrolled (Fig 1). Baseline characteristics are shown in Table 1. Mean age was 62.7 years and 56% was male. Median time between onset of symptoms and presentation at the ED was 3.0 hours. ACS was diagnosed in 149 (33%) patients: 13 (3%) STEMI, 104 (23%) NSTEMI, and 32 (7%) UA. The non-ACS group consisted of 304 individuals with a final diagnosis of stable angina (n = 48), rhythm disorders (n = 14), heart failure (n = 4), pericarditis (n = 1) or non-cardiac diagnoses (n = 237; e.g. aspecific chest pain, gastroesophageal reflux disease, myalgic chest pain). Patients who presented at the ED within three hours, were similar to the overall group, except for smoking, history of MI, PCI or CABG, and hypertension. In these patients, ACS was diagnosed in 67 (34%) cases. The completeness of the data for each biomarker is as follows: hs-cTnT 100%, PlGF 99.8%, sFlt-1 99.1%, myoglobin 100%, NT-proBNP 99.8%, GDF-15 99.8%, copeptin 68.2%.

**Table 1:** Baseline characteristics stratified by time of presentation after symptom onset.

Characteristics	N	All patients	N	Patients within 3h of symptom onset (n = 197)
Age, mean years	453	62.6 ± 14.5	197	61.8 ± 15.1
Male gender	453	253 (56%)	197	108 (55%)
Duration of symptoms in hours, median (IQR)	430	3.0 (1.8–6.8)	197	1.6 (1.2–2.2)
Hypertension	447	193 (43%)	194	68 (35%)
Hypercholesterolemia	447	148 (33%)	194	63 (33%)
Diabetes mellitus	447	72 (16%)	193	27 (14%)
Current smoker	444	114 (26%)	192	58 (30%)
Former smoker	444	111 (25%)	192	51 (27%)
Family history of CVD	442	181 (41%)	190	75 (40%)
BMI, mean kg/m²	320	27.0 ± 4.7	139	26.5 ± 4.5
Previous CVA	447	7 (2%)	194	3 (2%)
Previous TIA	447	22 (5%)	194	7 (4%)
Previous MI	446	96 (22%)	193	50 (26%)
Previous PCI	447	97 (22%)	194	48 (25%)
Previous CABG	446	45 (10%)	193	19 (10%)
Any MI, PCI or CABG	450	150 (33%)	196	73 (37%)
Heart failure	448	24 (5%)	194	12 (6%)
Peripheral arterial disease	447	25 (6%)	194	14 (7%)
Current aspirin use	440	187 (43%)	192	84 (44%)
Current clopidogrel use	436	50 (12%)	190	23 (12%)
Current coumarin use	436	47 (11%)	190	18 (10%)
Current ß-blocker use	437	171 (39%)	191	75 (39%)
Current statin use	439	176 (40%)	131	83 (44%)
Outcome of ACS	453	149 (33%)	197	67 (34%)
- STEMI		- 13 (3%)		- 7 (4%)
- NSTEMI		- 104 (23%)		- 43 (22%)
- UA		- 32 (7%)		- 17 (9%)

Values are given as mean (±Standard Deviation), median (IQR = Inter Quartile Range) or proportion (%)

Abbreviations: CVD, cardiovascular disease; BMI, body mass index; CVA, cerebrovascular accident; TIA, transient ischemic attack; MI, myocardial infarction; PCI, percutaneous coronary intervention; CABG, coronary artery bypass graft.

### Univariable analysis

Median levels of hs-cTnT, PlGF, sFlt-1, myoglobin, NT-proBNP, GDF-15 and copeptin were higher in ACS patients than in non-ACS patients (Table 2). Hs-cTnT had the largest AUC (0.86, 95% confidence interval (CI) 0.81–0.91) (Table 3). Myoglobin and NT-proBNP each had an AUC of 0.75 (95% CI 0.69–0.81), while the AUCs for PlGF, sFlt-1, GDF-15 and copeptin were lower. Of all biomarkers, hs-cTnT had both the highest positive predictive value (PPV) and negative predictive value (NPV): 77.9% and 86.4% respectively (Table 3). Also in the group of patients presenting within three hours, hs-cTnT still had the highest PPV and NPV (Table 4). On average, the PPVs increased and the NPVs decreased compared to the overall group. Strong clinical predictors for the presence of ACS were age (OR 1.05 per year), male gender (OR 1.63), a history of hypertension (OR 2.24), hypercholesterolemia (OR 1.71) or heart failure (OR 3.99), MI on ECG (OR 5.31) or ischemic ECG (OR 7.87) and the use of aspirin (OR 1.70), clopidogrel (OR 2.53) and β-blocker (OR 1.84) (Table 5).

**Table 2:** Median biomarker concentrations and inter quartile ranges stratified by ACS status.

	ACS		
Marker	Yes	No	p-value
	n = 149	n = 304	
hs-cTnT (pg/mL)	25.2 (11.7–81.1)	3.3 (1.2–7.7)	<0.001
PlGF (pg/mL)	17.3 (13.6–20.0)	14.0 (11.2–17.1)	<0.001
sFlt-1 (pg/mL)	69.7 (61.4–79.5)	63.3 (55.6–72.2)	<0.001
Myoglobin (ng/mL)	56.2 (40.2–121.4)	37.1 (29.1–48.8)	<0.001
NT-proBNP (pg/mL)	330.3 (118.8–1245.8)	78.8 (30.7–207.6)	<0.001
GDF-15 (pg/mL)	1221.0 (914.1–2160.7)	884.3 (672.5–1307.4)	<0.001
Copeptin (pmol/L)	9.2 (1.0–29.5)	6.2 (1.0–14.1)	0.005

Values are given as median (Inter Quartile Range); p-value calculated with Mann-Whitney *U*-test

Abbreviations: hs-cTnT, high-sensitive cardiac troponin; PlGF, placental growth factor; sFlt-1, soluble Fms-like tyrosine kinase-1; NT-proBNP, N-terminal prohormone B-type Natriuretic Peptide; GDF-15, growth differentiation factor-15.

**Table 3:** Sensitivity, specificity, predictive values and AUCs of hs-cTnT, myoglobin and 5 novel biomarkers in all patients.

	All patients (n = 453)				
Marker	Sensitivity	Specificity	PPV	NPV	AUC
hs-cTnT	71.1% (63.8–78.4)	90.1% (86.8–93.5)	77.9% (71.0–84.9)	86.4% (82.7–90.2)	0.86 (0.81–0.91)
PlGF	2.7% (0.1–5.3)	95.7% (93.4–98.0)	23.5% (3.4–43.7)	67.0% (62.5–71.3)	0.68 (0.62–0.74)
sFlt-1	47.3% (39.2–55.4)	72.3% (67.2–77.3)	45.1% (37.2–53.0)	74.0% (69.0–79.0)	0.62 (0.56–0.69)
Myoglobin	59.7% (51.9–67.6)	76.3% (71.5–81.1)	55.3% (47.6–63.0)	79.5% (74.8–84.1)	0.75 (0.69–0.81)
NT-proBNP	73.8% (66.8–80.9)	61.4% (55.9–66.9)	48.5% (42.0–55.0)	82.7% (77.7–87.6)	0.73 (0.67–0.79)
GDF-15	34.9% (27.2–42.6)	87.1% (83.4–90.9)	57.1% (47.0–67.3)	73.1% (68.6–77.7)	0.66 (0.59–0.72)
Copeptin	38.6% (29.1–48.1)	75.0% (69.1–80.9)	42.9% (32.7–53.0)	71.6% (65.6–77.5)	0.60 (0.53–0.67)

Values are given as percentage or number (95%CI)

Abbreviations: hs-cTnT, high-sensitive cardiac troponin; PlGF, placental growth factor; sFlt-1, soluble Fms-like tyrosine kinase-1; NT-proBNP, N-terminal prohormone B-type Natriuretic Peptide; GDF-15, growth differentiation factor-15; PPV, positive predictive value; NPV, negative predictive value; AUC, area under the (receiver operating?) curve.

**Table 4:** Sensitivity, specificity, predictive values and AUCs of hs-cTnT, myoglobin and 5 novel biomarkers in patients with symptom onset within 3 hours.

	Patients with symptom onset <3h (n = 197)				
Marker	Sensitivity	Specificity	PPV	NPV	AUC
hs-cTnT	62.7% (51.1–74.3)	92.3% (87.7–96.9)	80.8% (70.1–91.5)	82.8% (76.6–88.9)	0.86 (79.0–92.8)
PlGF	3.0% (0.0–7.1)	97.7% (95.1–100)	40.0% (0.0–82.9)	66.1% (59.5–73.0)	0.71 (62.1–80.2)
sFlt-1	43.3% (31.4–55.1)	77.5% (70.3–84.7)	50.0% (37.1–62.9)	72.5% (65.0–79.9)	0.62 (52.2–71.9)
Myoglobin	55.2% (43.3–67.1)	78.5% (71.4–85.5)	56.9% (44.9–69.0)	77.3% (70.1–84.4)	0.76 (67.0–84.0)
NT-proBNP	68.7% (57.5–79.8)	66.7% (58.5–74.8)	51.7% (41.3–62.1)	80.4% (72.8–87.9)	0.74 (65.1–82.9)
GDF-15	34.3% (65.1–82.9)	89.2% (83.9–94.6)	62.2% (46.5–77.8)	72.5% (65.6–79.4)	0.66 (56.1–76.1)
Copeptin	39.6% (25.7–53.4)	69.4% (59.6–79.2)	42.2% (27.8–56.7)	67.0% (57.2–76.9)	0.57 (46.6–67.4)

Values are given as percentage or number (95%CI)

Abbreviations: hs-cTnT, high-sensitive cardiac troponin; PlGF, placental growth factor; sFlt-1, soluble Fms-like tyrosine kinase-1; NT-proBNP, N-terminal prohormone B-type Natriuretic Peptide; GDF-15, growth differentiation factor-15; PPV, positive predictive value; NPV, negative predictive value; AUC, area under the receiver operating curve (ROC).

**Table 5:** Univariable analysis of possible predictors.

	Predictor	ACS	Non-ACS	Odds Ratio	95% CI
		n = 149	n = 304		
Risk factors	Age	69.0±13.2	59.5±14.1	1.05	1.04–1.07
	Male gender	95 (63.8%)	158 (52.0%)	1.63	1.09–2.43
	Hypertension	86 (57.7%)	112 (36.8%)	2.24	1.50–3.36
	Hypercholesterolemia	61 (40.9%)	90 (29.6%)	1.71	1.13–2.59
	Diabetes mellitus	29 (19.5%)	44 (14.5%)	1.46	0.87–2.45
	Current smoker	37 (24.8%)	77 (25.3%)	0.96	0.59–1.57
	Former smoker	38 (25.5%)	81 (26.6%)	0.92	0.57–1.50
	Family history of CVD	67 (45.0%)	121 (39.8%)	1.21	0.81–1.81
History	Previous CVA	6 (4.0%)	5 (1.6%)	2.23	0.57–8.76
	Previous TIA	8 (5.4%)	14 (4.6%)	1.30	0.53–3.21
	Previous MI	45 (30.2%)	55 (18.1%)	1.98	1.25–3.13
	Previous PCI	36 (24.2%)	62 (20.4%)	1.29	0.81–2.06
	Previous CABG	21 (14.1%)	25 (8.2%)	1.80	0.97–3.35
	Any MI, PCI or CABG	64 (43.0%)	87 (28.6%)	1.86	1.24–2.81
	Heart failure	16 (10.7%)	9 (3.0%)	3.99	1.69–9.43
	Peripheral arterial disease	15 (10.1%)	14 (4.6%)	2.21	1.00–4.90
Medication	Current aspirin use	76 (51.0%)	115 (37.8%)	1.70	1.14–2.53
	Current clopidogrel use	28 (18.8%)	25 (8.2%)	2.53	1.41–4.54
	Current coumarin use	21 (14.1%)	27 (8.9%)	1.74	0.95–3.18
	Current β-inhibitor use	73 (49.0%)	104 (34.2%)	1.84	1.23–2.75
	Current statin use	66 (44.3%)	112 (36.8%)	1.34	0.89–2.01
ECG	Acute MI on ECG	18 (12.1%)	8 (2.6%)	5.31	0.81–34.84
	Ischemic ECG	103 (69.1%)	66 (21.7%)	7.87	4.96–12.48

Values are given as mean (±SD) or proportion (%)

Abbreviations: CVD, cardiovascular disease; CVA, cerebrovascular accident; TIA, transient ischemic attack; MI, myocardial infarction; PCI, percutaneous coronary intervention; CABG, coronary artery bypass graft; MI, myocardial infarction; ECG, electrocardiogram; ACS, acute coronary syndrome; CI, confidence interval.

### Multivariable analysis

**Fig 2:**
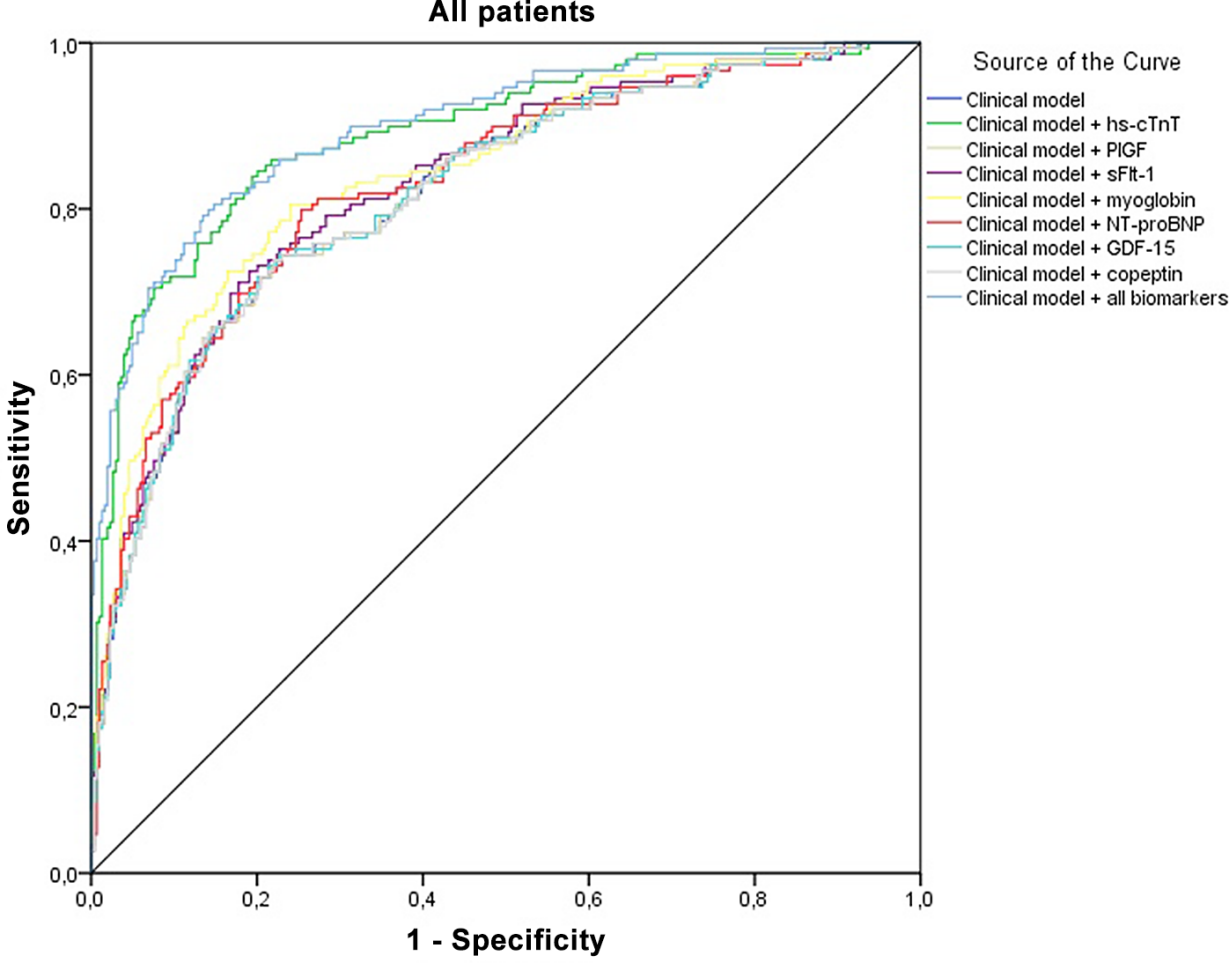
Receiver-operating-characteristic curves of the clinical model with the various biomarkers, high sensitive cardiac troponin T (hs-cTnT), placental growth factor (PlGF), fms-like tyrosine kinase-1 (sFlt-1), myoglobin, N-terminal prohormone B-type Natriuretic Peptide (NT-proBNP), growth differentiation factor-15 (GDF-15) and copeptin. (The ROC curve shown is from the first imputation set)

The clinical model with age, sex, history of MI, PCI or CABG, cardiovascular risk factors and ECG features resulted in an AUC of 0.80 (Table 6, Fig 2). Addition of hs-cTnT to this model resulted in the most profound increase in the AUC (0.89; Likelihood ratio test (LR test) p<0.001). Only addition of myoglobin to the clinical model showed a small (significant) increase in the AUC of 0.84. Addition of any of the novel biomarkers to the clinical model and hs-cTnT levels did not or only marginally increase the AUC (Table 6; all AUCs 0.88–0.90), although adding GDF-15 (significantly) improved calibration (LR test p = 0.026). Combining all the biomarkers with the clinical model did not result in an increase in AUC (0.89). Similar results were observed in patients presenting to the ED <3 hours (Table 7). Adding hs-cTnT to the clinical model resulted in the highest increase in AUC (0.88, LR test p<0.001). None of the other biomarkers yielded diagnostic information in addition to the clinical model and hs-cTnT levels, with the exception of copeptin, which showed an AUC of 0.89 (non-significant).

**Table 6:** Multivariable analysis including all patients (n = 453).

Model	AUC*	95% CI	Likelihood ratio test
			p-value
Clinical model	0.80	0.76–0.84	
*Clinical model with hs-cTnT*	0.89	0.87–0.94	p<0.001**
Clinical model with PlGF	0.81	0.77–0.85	p = 0.887**
Clinical model with sFlt-1	0.82	0.78–0.86	p = 0.001**
Clinical model with myoglobin	0.84	0.80–0.88	p<0.001**
Clinical model with NT-proBNP	0.82	0.79–0.87	p<0.001**
Clinical model with GDF-15	0.81	0.77–0.85	p = 0.664**
Clinical model with copeptin	0.81	0.77–0.85	p = 0.683**
*Clinical model*, *hs-cTnT* and PlGF	0.88	0.86–0.93	p = 0.081***
*Clinical model*, *hs-cTnT* and sFlt-1	0.88	0.86–0.92	p = 0.892***
*Clinical model*, *hs-cTnT* and myoglobin	0.88	0.86–0.93	p = 0.693***
*Clinical model*, *hs-cTnT* and NT-proBNP	0.88	0.86–0.92	p = 0.216***
*Clinical model*, *hs-cTnT* and GDF-15	0.90	0.87–0.94	p = 0.026***
*Clinical model*, *hs-cTnT* and copeptin	0.88	0.86–0.92	p = 0.315***
Clinical model with *all* biomarkers	0.89	0.87–0.93	p<0.001**
			p = 0.191***

Clinical model: Age, sex, hypertension, hypercholesterolemia, family history of CVD, current and former smoking, diabetes mellitus, and history of MI, PCI or CABG and ECG. Abbreviations: hs-cTnT, high-sensitive cardiac troponin; PlGF, placental growth factor; sFlt-1, soluble Fms-like tyrosine kinase-1; NT-proBNP, N-terminal prohormone B-type Natriuretic Peptide; GDF-15, growth differentiation factor-15; AUC, area under the receiver operating curve (ROC); CI, confidence interval.

*adjusted for over-optimism

** compared to the Clinical model

*** compared to the Clinical model + hs-cTnT

**Table 7:** Multivariable analysis including patients within 3 hours of symptom onset (n = 197).

Model	AUC*	95% CI	Likelihood ratio test
			p-value
Clinical model	0.81	0.75–0.87	
*Clinical model with hs-cTnT*	0.88	0.84–0.94	p<0.001**
Clinical model with PlGF	0.82	0.76–0.88	p = 0.519**
Clinical model with sFlt-1	0.82	0.76–0.88	p = 0.068**
Clinical model with myoglobin	0.83	0.77–0.89	p = 0.015**
Clinical model with NT-proBNP	0.84	0.78–0.90	p = 0.003**
Clinical model with GDF-15	0.82	0.76–0.88	p = 0.800**
Clinical model with copeptin	0.82	0.76–0.88	p = 0.355**
*Clinical model*, *hs-cTnT* and PlGF	0.88	0.85–0.94	p = 0.470***
*Clinical model*, *hs-cTnT* and sFlt-1	0.87	0.84–0.93	p = 0.688***
*Clinical model*, *hs-cTnT* and myoglobin	0.88	0.85–0.94	p = 0.766***
*Clinical model*, *hs-cTnT* and NT-proBNP	0.88	0.84–0.94	p = 0.404***
*Clinical model*, *hs-cTnT* and GDF-15	0.88	0.85–0.94	p = 0.182***
*Clinical model*, *hs-cTnT* and copeptin	0.89	0.85–0.94	p = 0.169***
Clinical model with *all* biomarkers	0.89	0.85–0.94	p<0.001**
			p = 0.304***

Clinical model: Age, sex, hypertension, hypercholesterolemia, family history of CVD, current and former smoking, diabetes mellitus, and history of MI, PCI or CABG, and ECG. Abbreviations: hs-cTnT, high-sensitive cardiac troponin; PlGF, placental growth factor; sFlt-1, soluble Fms-like tyrosine kinase-1; NT-proBNP, N-terminal prohormone B-type Natriuretic Peptide; GDF-15, growth differentiation factor-15; AUC, area under the receiver operating curve (ROC); CI, confidence interval.

*adjusted for over-optimism

** compared to the Clinical model

*** compared to the Clinical model + hs-cTnT

## Discussion

In patients suspected of ACS, high-sensitive troponin assays are not always conclusive in the first hours after symptom onset, and so the search for novel early biomarkers is ongoing. This prospective study assessed the diagnostic value of several novel biomarkers in combination with the patient’s history, cardiovascular risk factors and ECG findings, in diagnosing ACS at an early stage. Our results show that hs-cTnT is still the best biomarker when trying to determine the presence of ACS, both in a single marker diagnosis and when integrated into our clinical model (AUCs of respective 0.86 and 0.89). The biomarker myoglobin provided additional value to the clinical model, but not when hs-cTnT was added to the clinical model (AUC 0.88). The other biomarkers studied provided no additional diagnostic information to the clinical model.

We compared our results with those from other recent biomarker studies. Firstly, copeptin has been extensively investigated as a possible addition to hs-cTn, with several recent studies presenting promising results. Meune et al. [8] measured hs-cTnT and copeptin in a comparable study population with the same cut-off values for the biomarkers. They found a NPV of 82.6% when combining copeptin with hs-cTnT on admission, and an AUC of 0.94, compared to a NPV of 76.5% and an AUC of 0.90 for hs-cTnT on admission alone (non-significant difference). This apparent advantage of using copeptin is diminished when looking at hs-cTnT values at three hours after admission. They show a NPV of 83.9% and an AUC of 0.94. Maisel et al.[9] showed in a recent large trial that adding copeptin to cTnI allowed safe rule out of AMI with a NPV of 99%, promoting a multimarker approach, whereas our study did not show a significant added value of copeptin. Möckel et al. [29] concluded in a RCT on 902 patients that a single measurement of troponin and copeptin allows for early discharge of low- to intermediate risk patients with suspected ACS and seems to be safe. However, mentioned studies did not incorporate a clinical model in their studies, making an assessment of the added value of copeptin to clinical characteristics impossible. Furthermore, earlier studies that did find a significant advantage of using copeptin for early diagnosis or exclusion mostly used conventional troponin assays, instead of high sensitive troponin assays, or included patients with STEMIs.[17, 23] Secondly, similar to our findings, Schaub et al. showed there is little value in using GDF-15 as a diagnostic test in chest pain patients. GDF-15 seems more valuable as a prognostic marker.[10, 18] Thirdly, PlGF and NT-proBNP have also been previously investigated. In a study where both markers are explored in a single marker strategy, PlGF has a sensitivity of 24%, a specificity of 70% and an AUC of 0.50,[13] while the corresponding findings in our study were 2.7%, 95.7% and 0.68 respectively. Although these values differ considerably, both studies concluded that PlGF is not suitable for a single marker strategy. Their results for NT-proBNP also differed from ours, but to a lesser extent. When added to conventional troponin I, PlGF and NT-proBNP did not provide any clinically significant additional diagnostic value in their study; a finding confirmed in our study. Lastly, when comparing hs-cTnT with myoglobin, our results are in line with other studies as well (Kurz et al,[30]).

Half of the studies mentioned above used the standard, non-high-sensitive troponin assays, whereas one of the strengths of our study is the use of a high-sensitive assay. Earlier studies confirmed the higher sensitivity of the so-called high sensitivity troponin assays compared to conventional assays.[5, 6] However, higher sensitivity is usually accompanied by lower specificity and concerns have been raised about the number of false-positives.[8, 19] Mechanisms other than coronary artery plaque rupture (main cause of type 1 myocardial infarction) can cause myocardial injury and result in elevation of troponin, like heart failure, renal failure and sepsis. These so called type 2 myocardial infarctions result from an imbalance between myocardial oxygen supply and/or demand, other examples of which are coronary vasospasm, anaemia and hypotension.[4, 13, 19, 31]

As the search for the perfect biomarker continues, many researchers support a multimarker approach in diagnosing ACS.[29, 32] Such an approach is not only advocated to be able to diagnose ACS quickly, but also to find a cause for the elevated troponin levels in other heart diseases and non-cardiac diseases.[19] A multimarker strategy combining cardiac troponin with other markers of myocardial damage, or biomarkers “upstream” from necrosis, may help to gain insight into the pathophysiological mechanisms causing non-ACS related troponin leakage.[10]

We aimed to develop such a multimarker strategy with the aid of some biomarkers often advocated as useful adjuncts to hs-cTn, but were unsuccessful. None of these added relevant diagnostic information to a clinical model plus hs-cTnT.

Previous studies investigating novel biomarkers predominantly focused on myocardial infarctions as the primary outcome.[13, 17, 19] However, patients with unstable angina are at a clearly increased risk of adverse cardiovascular events.[1, 2] Recognizing these patients early and treating them accordingly is likely to improve prognosis. We therefore chose to include UA in our outcome. Unfortunately, subgroup analyses comparing patients with unstable angina versus “no ACS” revealed no additional value of the novel biomarkers compared with the clinical model alone (AUC of the clinical model 0.79 versus AUC of clinical model with single novel biomarker ranging from 0.76 to 0.79). It should be emphasized, however, that the number of UA patients is small. These findings should therefore be interpreted with caution.

One of the strengths of our study is the manner in which we conducted our data analysis to enable us to quantify the additional value of the biomarkers. We applied multiple imputation in case of missing values, and performed multivariable regression analysis to assess the value of the various biomarkers in combination with a patient’s history and ECG.

We recognize that our study has several limitations. Firstly, we used panel diagnosis for final adjudication of our outcome. However, in the absence of a reference standard, there is no alternative when one is interested in a clinically relevant outcome, furthermore, expert panels are widely accepted.[33, 34] Secondly, we only have single measurements of hs-cTnT, instead of serial measurements. Moreover, these measurements were assessed retrospectively. Thirdly, the completeness of the data for each biomarker was ranging from 99.1–100% in all biomarkers, except for copeptin, with complete data in 68.2%. Because we decided to investigate copeptin after the first analysis of the frozen samples had been done, we had a relative high number of missing values for copeptin, since for a number of participants no frozen samples were available. In our analyses we used multiple imputation to counteract this deficit. Moreover, the availability of remaining blood samples is very likely to be a random phenomenon and unrelated to the patients characteristics or outcome. Fourthly, we used one cut-off value for each biomarker, based on the available literature or clinical grounds. Theoretically, performance of these biomarkers could improve by using either a lower or higher cut-off value to detect ACS. Sensitivity analyses applying other cut-off points, however, did not improve diagnostic value of the markers. Fifthly, due to the observational nature of our study, we cannot provide any data on the possible effect of the use of these biomarkers on the patients’ prognosis, but such an effect is likely to be very limited in view of the minimal diagnostic yield of adding the biomarkers. Lastly, we used our prediction model on both our entire population and on the subgroup of patients presenting within 3 hours. The low number of events in combination with the number of predictors in our model could induce overfitting.[35, 36]

In conclusion, of the biomarkers tested, only the use of myoglobin had additional value to our clinical model in patients suspected of ACS. However, hs-cTnT was superior to all other biomarkers when used with our clinical model as well as in a single marker strategy and none of the other biomarkers provided significant diagnostic information in addition to the clinical model and hs-cTn. Research on the added value of novel biomarkers to complement troponin and clinical assessment should continue to further limit the number of false-positives and false-negatives.

## Supporting Information

S1 Text Measurement of biomarkers. (DOCX)
