# Supplementary material for: No Added Value of Novel Biomarkers in the Diagnostic Assessment of Patients Suspected of Acute Coronary Syndrome
Source: PLoS One. 2015 Jul 15;10(7):e0132000. doi: 10.1371/journal.pone.0132000 (PMC4503345; doi:10.1371/journal.pone.0132000)
Supplement: S1 Text — (DOCX) [file pone.0132000.s001.docx]

**S1 Text Measurement of biomarkers**

Hs-cTnT was measured with the Elecsys troponin T hs assay fourth generation (Roche Diagnostics) with a lower detecting limit of 3pg/mL. The 99^th^-percentage cut-off point was ≥14pg/mL. The coefficient of variation (CV) was <10% at 13 pg/mL. PlGF was measured with the Elecsys PlGF assay (Roche Diagnostics) with a measuring range 3-1000pg/mL. The CV was <5% for measured values. sFlt-1 was measured with the Elecsys sFlt-1 assay (Roche Diagnostics) with a measuring range of 10-85000pg/ml. The CV was <5% for measured values. Myoglobin was measured with the Elecsys myoglobin assay (Roche Diagnostics) with a measuring range 21-3000ng/mL. The CV was <10% for all levels. NT-proBNP was measured with the Elecsys proBNP II assay (Roche Diagnostics, Switzerland). The lower detection limit was 5pg/mL and the CV was <5% for measured values. GDF-15 was measured with the GDF15 sandwich immunoradiometric sandwich assay. The lower detection limit was 20ng/L. The intra assay imprecision ranged from 2.85 to 10.6% and the inter assay imprecision ranged from 4.05 to 12.2%. Copeptin was measured with the commercial sandwich immunoluminometric assay (B.R.A.H.M.S. LUMItest CT-proAVP, B.R.A.H.M.S AG, Hennigsdorf/Berlin, Germany). The lower detection limit was 0.4pmol/l, and the functional assay sensitivity (<20% interassay CV) was <1pmol/l. If the measured copeptin level was ‘low’ we used 1pmol/L.
